# Yield and clinical impact of image-guided bone biopsy in osteomyelitis of the appendicular skeleton: a systematic review and meta-analysis

**DOI:** 10.1007/s00256-024-04764-7

**Published:** 2024-07-30

**Authors:** Karen Smayra, Shahid Miangul, Maria M. Witkowiak, Linn K. M. Persson, Emily E. Lugard, Maamoun Adra, Nathanael Q. E. Yap, Jake Ball, Hayato Nakanishi, Christian A. Than, Michael Khoo

**Affiliations:** 1 St George’s University of London, London, SW17 0RE UK; 2 University of Nicosia Medical School, University of Nicosia, 2417 Nicosia, Cyprus; 3 Department of Medical Imaging, John Hunter Hospital, Newcastle, Australia; 4 Faculty of Health, University of Newcastle, Newcastle, Australia; 5 School of Biomedical Sciences, The University of Queensland, St Lucia, Brisbane, QLD 4072 Australia; 6 Department of Radiology, Royal National Orthopaedic Hospital, Stanmore, HA7 4LP UK

**Keywords:** Image-guided, Osteomyelitis, Biopsy, Appendicular skeleton

## Abstract

**Objective:**

To assess the yield and clinical impact of image-guided bone biopsy for osteomyelitis of the appendicular skeleton.

**Materials and methods:**

A literature search of several databases was conducted from inception to August 2023. Eligible studies reported patients who underwent image-guided bone biopsy for investigation of osteomyelitis of the appendicular skeleton. The pooled proportions were analyzed using a random-effects model. This review was registered in PROSPERO (CRD42023466419).

**Results:**

From 370 initial studies screened, eight met the eligibility criteria, with a total of 700 patients. The pooled technical success rate was 99.6% (95% CI: 0.992, 1.001; *I*^*2*^ = 0%). Positive bone cultures were pooled at 31.9% (95% CI: 0.222, 0.416; *I*^*2*^ = 87.83%) and negative cultures at 68.1% (95% CI: 0.584, 0.778; *I*^*2*^ = 87.83%). Methicillin-Sensitive Staphylococcus Aureus and Methicillin-Resistant Staphylococcus Aureus yield was 24.5% (95% CI: 0.096, 0.394; *I*^*2*^ = 90.98%) and 7.6% (95% CI: 0.031, 0.121; *I*^*2*^ = 34.42%) respectively. Group A Streptococcus yield was 7.0% (95% CI: 0.014, 0.127; *I*^*2*^ = 70.94%). Polymicrobial culture yield was 15.7% (95% CI: 0.018, 0.297; *I*^*2*^ = 88.90%). Post-procedural management change rate was 36.5% (95% CI: 0.225, 0.504; *I*^*2*^ = 92.39%). No complications were reported across studies.

**Conclusion:**

For patients under investigation of osteomyelitis of the appendicular skeleton, image-guided bone biopsy demonstrates a good rate of technical success. Additional studies may provide further support for the use of image-guided bone biopsy in this population. Image-guided bone biopsy results lead to change in antibiotics therapy in a portion of patients with suspected osteomyelitis suggesting its potential utility in select patients.

**Supplementary Information:**

The online version contains supplementary material available at 10.1007/s00256-024-04764-7.

## Introduction

Osteomyelitis (OM) is a debilitating infection of the bone that poses a significant challenge in clinical diagnosis, particularly when affecting the appendicular skeleton [1]. The precise incidence of OM in the United States remains uncertain, but reports suggest numbers as high as 1 in 675 hospital admissions annually, totaling approximately 50,000 cases. The condition is more common in men and higher age, which can be attributed to comorbid factors like diabetes mellitus (DM) and peripheral vascular disease [2].

The intricate nature of bone infections demands precise and reliable diagnostic approaches to facilitate timely intervention, accurate antimicrobial therapy and enhance patient outcomes. Radiography serves as the primary screening tool for clinically suspected OM [3]. However, it may not definitively exclude osteomyelitis, as imaging abnormalities can take up to 2 weeks in children and longer in adults to manifest. Nevertheless, X-rays play a crucial role in eliminating alternative diagnoses, such as fractures and malignancies [4]. Alternatively, Magnetic Resonance Imaging (MRI) stands out as the definitive diagnostic examination, offering both high sensitivity (78–90%) and specificity (60–90%), along with exceptional anatomical clarity [3]. The drawback of imaging modalities however, is the inability to provide information regarding the microbial aetiology [5].

In recent years, image-guided bone biopsy (IGBB) has emerged as a promising adjunctive diagnostic tool for OM [3]. This technique enables direct sampling of affected bone tissue, allowing for determination of the causative microorganisms and their antibiotic susceptibilities. This is critical, as intervention with extended antibiotic regimens and surgical removal of infected tissues may still result in persistent infection or additional disease-related complications [4]. By complementing conventional imaging, IGBB holds the potential to evolve the diagnostic landscape of OM. Previous meta-analyses by Pupaibool et al. (2015) and Schechter et al. (2020) synthesized the available literature on OM of the spine and diabetic foot respectively [6, 7]. Pupaibool et al. (2015) recorded a pooled sensitivity of 52.2% (95% CI: 0.458, 0.585; *I*^*2*^ = 83.8%), and a specificity of 99.9% (95% CI: 0.945, 1.00; *I*^*2*^ = 0%) for IGBB in vertebral OM [6]. However, the data concerning non-vertebral OM remains limited.

This systematic review and meta-analysis aim to comprehensively assess the existing body of literature on IGBB in the context of OM affecting the appendicular skeleton. By synthesizing and analyzing the available evidence, this study seeks to provide a thorough understanding of the yield and clinical utility of IGBB with the goal of refining diagnostic protocols and treatment strategies and ultimately improving the management of OM in the appendicular skeleton.

## Material and methods

### Search strategy and data sources

In adherence to the Preferred Reporting Items for Systematic Reviews and Meta-analyses (PRISMA) guidelines [8], an exhaustive exploration of various databases spanning from their inception to August 2023 was carried out. The databases encompassed PubMed, EMBASE (Elsevier), CiNAHL, Cochrane Central Register of Controlled Trials, Cochrane Database of Systematic Reviews, Scopus, and Web of Science. The search approach was formulated and executed by a medical librarian, in consultation with the principal investigator of the study. A combination of controlled vocabulary and keywords was employed to investigate topics related to IGBB in OM of the appendicular skeleton. The precise details of the search strategy, including all terms used and their combinations, can be found in Supplementary Fig. A. This review was prospectively registered with PROSPERO (CRD42023466419).

### Eligibility criteria and quality assessment

Qualifying studies were required to satisfy the following inclusion criteria for participants: individuals of all age groups undergoing IGBB to investigate suspected OM, with a stipulation that ≥ 90% of the bone biopsies were derived from the appendicular skeleton. These studies were expected to provide information on the technical success rate of bone biopsies, the percentage of positive and negative bone cultures, and the percentage of samples with positive histological analysis for OM.

Excluded from consideration in this study were case reports, case series, abstracts, conference abstracts, reviews, and articles not reported in English. Notably, the meta-analysis did not impose any restrictions based on sample size. The screening of articles and extraction of data were independently carried out by three assessors (LKMP, MMW, EEL), with any discrepancies resolved by KS and discussed with co-authors as needed. The quality of each study was assed by KS and LKMP using the ROBINS-1 Tool [9]. In cases of disparity, two independent assessors deliberated, and disagreements were settled through adjudication by CAT.

### Extracted outcomes and definitions

Baseline (gender, weight, BMI), clinical (diagnosis, pre-operative therapy, laboratory and imaging investigations) and epidemiological (comorbidities) characteristics of the population, as well as outcomes pertaining to the technicalities of the procedure itself (level of expertise of the operator, needle type and gauge, intra-operative imaging, number and location of procedures, number of samples, aspiration yield), sampling results (culture yield, histological analysis) and follow-up (complications, mortality) were extracted. We defined technical success as the number of procedures through which adequate sampling was obtained and subsequently sent for culture with Gram staining and/or histopathological analysis. Change in management was defined as the number of patients for which there was a modification of the antibiotics therapy following biopsy results.

### Statistical analysis

The collective means and proportions of the data were examined employing a random-effects approach, specifically the generic inverse variance method introduced by DerSimonian and Laird. This method assigns weights to each study based on its variance [10]. Pooled proportions were computed on a per-lesion basis. To gauge the heterogeneity of effect size estimates across the studies, the Q statistic and I^2^ were utilized, with significance set at P < 0.10. I^2^ values were categorized as follows: 0–25% indicating insignificant statistical heterogeneity, 26–50% suggesting low heterogeneity, and 51–100% indicating high heterogeneity [11]. Additionally, a sensitivity analysis, known as leave-one-out, was conducted to evaluate the impact of each study on the pooled estimate. This involved omitting one study at a time and recalculating the combined estimates for the remaining studies. Publication bias was assessed through funnel plots generated through the jamovi software (The jamovi project (2024), jamovi (Version 2.5)) [12]. In instances where mean and standard deviation (SD) were not available, the median was converted to mean using formulas from the Cochrane Handbook for Systematic Reviews of Interventions [11]. The analysis of the data was carried out using the OpenMetaAnalyst software developed by the Center for Evidence-Based Medicine at Brown University, Providence, Rhode Island, USA.

## Results

### Study selection and patient characteristics

A comprehensive search of electronic databases identified 370 studies. After eliminating duplicates, a thorough screening of titles and abstracts was conducted, whilst adhering to specified inclusion and exclusion criteria. This process identified 20 full-text articles that were deemed eligible for further evaluation. Ultimately, eight studies [13–20] with a total of 700 patients, conducted between 2007 and 2023 met the criteria and were included in this quantitative meta-analysis. All the studies incorporated were retrospective, comprising seven single-centered studies [13–17, 19, 20] and one multi-centered study [18]. Among these, six studies [13–16, 18, 20] reported on 528 patients for which the mean age ranged from 10.7 to 64.5 years. Five of the included studies [13–16, 20] reported on the gender distribution of their population, of which 127 (26.1%) were female.

The study selection process is illustrated in the PRISMA flow chart (Supplementary Fig. B). All included studies adhered to the principles outlined in the Declaration of Helsinki. Comprehensive details of the baseline characteristics of the included studies are provided in Table 1.

**Table 1:** Baseline Characteristics of Included Studies

Study	Publication Year	Study type	Country	Total Number of Participants (n)	Gender, Male / Female, n (%)	Age, Mean ± SD (Years)
Al-Balas et al.	2023	Retrospective	USA	170	168 (98.8%) / 2 (1.2%)	64.5 ± 15.3
Beroukhim et al.	2019	Retrospective	USA	29	18 (62.1%) / 11 (37.9%)	39.9 ± 12.2
Gajewski et al.	2023	Retrospective, Comparative	USA	15	10 (66.7%) / 5 (33.3%)	10.7 ± 4.9
Hirschfeld et al.	2019	Retrospective, Cohort	USA	185	114 (61.6%) / 71 (38.4%)	57.8 ± 17.0
Mammarappallil et al.	2018	Retrospective	USA	88	50 (56.8%) / 38 (43.2%)	60.0 ± 13.2
Said et al.	2019	Retrospective	USA	59	NR	NR
Wu et al.	2007	Retrospective	USA	75	24 (58.5%) / 17 (41.5%) *	40.8 ± 19.7 *
Yen et al.	2022	Retrospective	USA	79	NR	NR

NR Not reported, n Number, SD Standard deviation

*the available data pertains exclusively to the patients with positive histology (N=41)

### Risk of *bias* and quality assessment

The results of the quality assessment of the included studies are summarized in Supplementary Fig. C. All the studies included were deemed adequate within the selection domain. The results of publication bias assessment and funnel plots are available in Supplementary Fig. D.

### Pre-procedural clinical characteristics

**Fig. 1:**
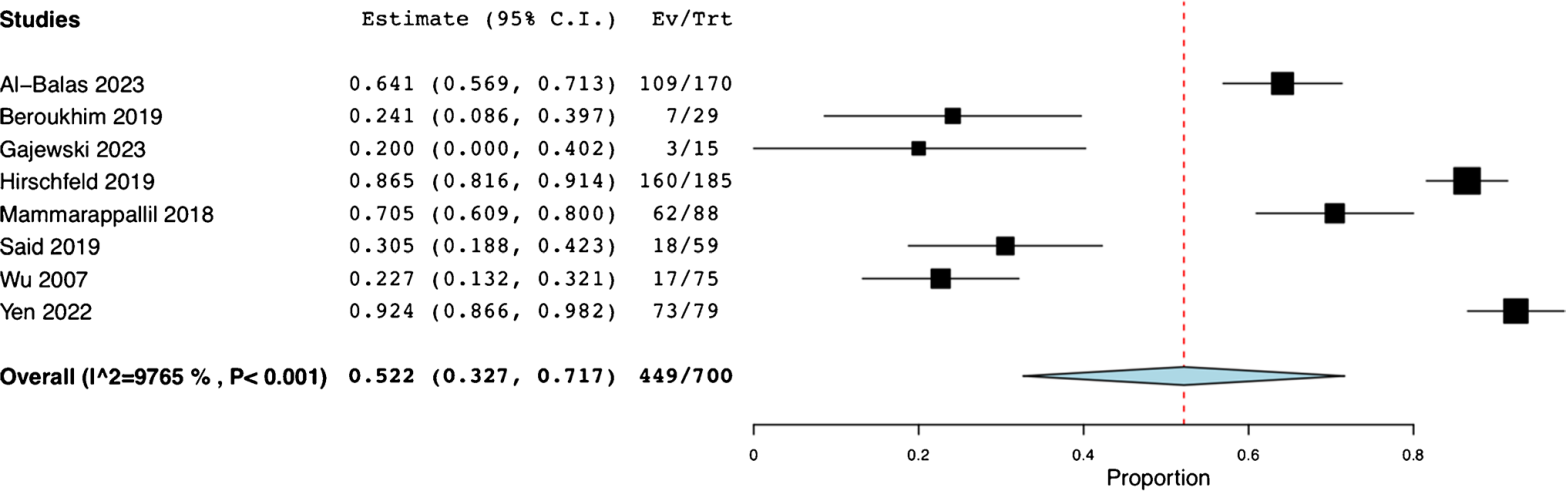
Pooled Rate of Pre-Procedural Antibiotics

In a collective analysis of eight studies, a total of 700 patients underwent image-guided bone biopsies. Within this cohort, one study [15] reported a mean body mass index of 18.7. Preceding the procedure, three studies [13, 14, 16] reported comorbidities of DM in 293 (76.3%) patients. Across the board, seven studies [13–19] collectively identified 598 patients as having suspected osteomyelitis before the procedure. One study [15] confirmed the diagnosis of acute osteomyelitis in 15 patients based on clinical findings. These included a combination of X-ray and MRI results as well as white blood cell count, erythrocyte sedimentation rate, and C-reactive protein levels. In one study [16], 155 cases of osteomyelitis were documented and verified through imaging, with MRI being the most frequently employed method. Similarly, in a separate study [20] there were 88 cases of MRI-diagnosed osteomyelitis. Before the bone biopsy, 52.2% of patients received antibiotics (95% CI: 0.327, 0.717; *I*^*2*^ = 97.65%) (N = 449) (Fig. 1) from all eight studies [13–20]. However, two studies [13, 17] reported that 67 patients discontinued their treatment 24 h prior to the procedure. Five studies [13, 16, 17, 19, 20] revealed that 450 (77.4%) patients underwent MRI, 291 (50.1%) underwent X-ray, 59 (10.1%) underwent a probe-to-bone assessment [13], and 44 (7.6%) underwent CT scans. In terms of microbiological evaluation, blood cultures were scrutinized in two studies [15, 19] uncovering positive results in 5 patients and negative results in 77 patients. Additionally, a separate study [17] reported one positive swab culture from a superficial wound foot. The data corresponding to clinical characteristics of the population are available in Table 2.

**Table 2:** Pre-procedural Characteristics

Study	Prevalence of Type 2 DM (n)	Pre-Procedural Diagnosis of OM (n)	Patients Under Pre-Procedural Antibiotics (n)	Pre-Procedural Imaging			Pre-Procedural Blood Culture, Positive / Negative (n)
				CT Scan (n)	MRI (n)	X-ray (n)	
Al-Balas et al.	170	170	109	0	74	136	NR
Beroukhim et al.	23	29	7	NR	NR	NR	NR
Gajewski et al.	NR	0	1	NR	NR	NR	1 / 13
Hirschfeld et al.	100	185	160	42	153	153	NR
Mammarappallil et al.	NR	NR	62	NR	88	NR	NR
Said et al.	NR	60	18	2	56	2	NR
Wu et al.	NR	75	17	NR	NR	NR	NR
Yen et al.	NR	79	73	NR	79	NR	4 / 64

*CT* Computed Tomography *DM* Diabetes mellitus, *MRI* Magnetic resonance imaging *NR* Not reported, *n* Number, *OM* Osteomyelitis

### Intraprocedural characteristics

A total of 635 procedures were performed out of 612 patients, as reported by seven studies [13–19]. One study [20] reported the number of samples obtained and not the number of procedures. A total of 1088 core samples were obtained across all studies [13–20]. Most samples were obtained from the bones of the foot, totaling 500 (45.9%). There were 143 (13.1%) samples taken from the pelvis, 91 (8.4%) from the tibia, 87 (8.0%) from the femur, 82 (7.5%) from the patella, 3 (0.3%) from the humerus, 3 (0.3%) from the clavicle, and 3 (0.3%) from the fibula. Additionally, 2 (0.2%) samples was taken from the sacrum, and in 174 (16.0%) instances, the location was not specified. One study [15] did not report the location of for any of their samples.

In total, four studies [13–15, 18] reported the modality used for intra-procedural imaging, with 183 (71.8%) individuals undergoing fluoroscopy and 72 (38.2%) undergoing CT scans. Of these, Wu et al. (2007) reported only the type of intra-procedural imaging within one group (N = 41) of their total population (N = 75) [18]. Three of the studies did not report the exact distribution for intra-procedural imaging [16, 17, 19]. Of these, Said et al. (2019) describe that biopsy specimens were typically obtained under CT guidance, with fluoroscopy guidance used for a small number of foot biopsies [17]. Yen et al. (2023) state that most intraprocedural imaging was done by fluoroscopy and US guidance [19]. The data pertaining to intraprocedural characteristics is summarized in Table 3.

**Table 3:** Intra-Procedural Characteristics

Study	Total Number of Procedures (n)	Number of Core Samples Obtained (n)	Location of Sampling (n)	Location of Sampling Not Specified (n)	Intra-Procedural Imaging	
					Fluoroscopy (n)	CT Scan (n)
Al-Balas et al.	170	171	Bones of the foot (170) Fibula (1)	0	170	0
Beroukhim et al.	29	29	Bones of the foot (2) Clavicle (2) Femur (10) Fibula (1) Humerus (1) Patella (1) Pelvis (7) Tibia (5)	0	0	29
Gajewski et al.	15	15	NR	15	0	15
Hirschfeld et al.	203	203	Bones of the foot (123) Pelvis (42)	38	NR	NR
Mammarappallil et al.	NR	88	Bones of the foot (86) Tibia (2)	0	NR	NR
Said et al.	62	60	Bones of the foot (25)	35	NR	NR
Wu et al.	75	36*	Bones of the foot (9) Clavicle (1) Humerus (2) Femur (4) Fibula (1) Pelvis (13) Sacrum (2)	4	13*	28*
Yen et al.	82	486	Bones of the foot (85) Femur (73) Patella (81) Pelvis (81) Tibia (84)	82	NR	NR

*NR* Not reported, *n* Number

*the available data pertains exclusively to the patients with positive histology (N=41)

Among the eight included studies, two [18, 20] report on needle type and gauge for biopsy sampling. The most used was the Bonopty® coaxial system (AprioMed), accounting for 163 cases. The needle gauge was reported for 126 procedures, with 18G being the most popular choice, used for 89 of these procedures (70.6%). For the remaining procedures, 17 (13.5%) used 15G, 17 (13.5%) used 14G, and 3 (2.4%) used 11G needles.

Only one paper reported on the quality of punctured skin [16]. In total, 150 samples were taken from a site with overlying ulcer, and 77 from sites with overlying cellulitis [16]. Two studies specified that infected or ulcerated skin was avoided while taking the biopsy [16, 17]. In contrast, one study did not specify if the avoidance of passage through ulceration was possible [13].

The level of expertise of the operator was mentioned in five studies [15, 17–20], and all of them reported that the procedures were conducted by a single or group of experienced radiologists.

### Post-procedural outcomes

**Fig. 2:**
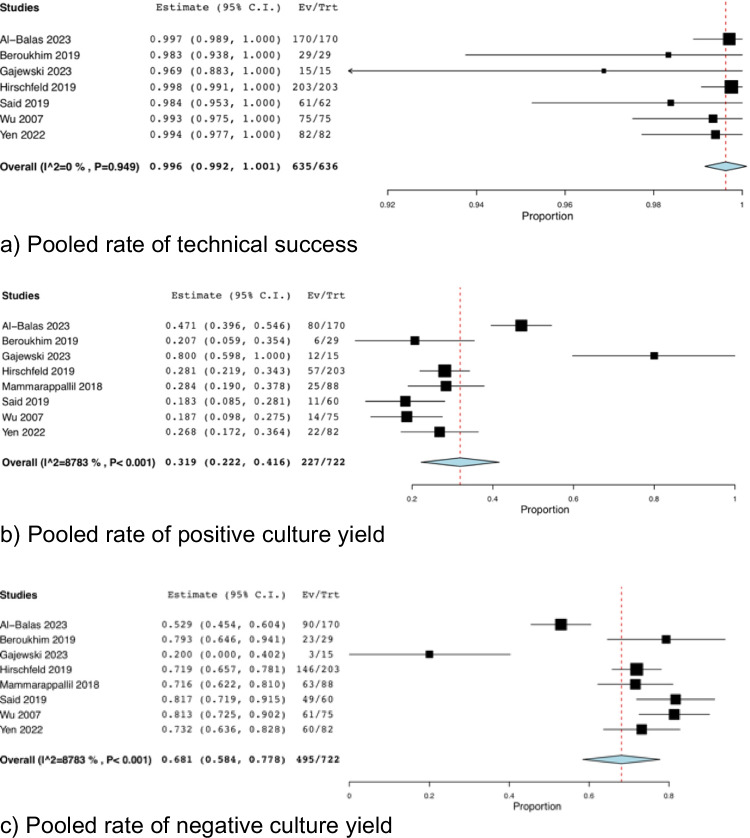
Technical and Culture Yield Outcomes (**a**) pooled rate of technical success (**b**) Pooled rate of positive culture yield (**c**) Pooled rate of negative culture yield

Seven studies reported on technical success with a total of 635 procedures of which 634 were deemed successful [13–19]. The pooled rate of technical success was evaluated to be 99.6% (95% CI: 0.992, 1.001; *I*^*2*^ = 0%) (Fig. 2a). Of these, there was one procedural failure attributed to the sample getting lost in transit [17].

**Fig. 3:**
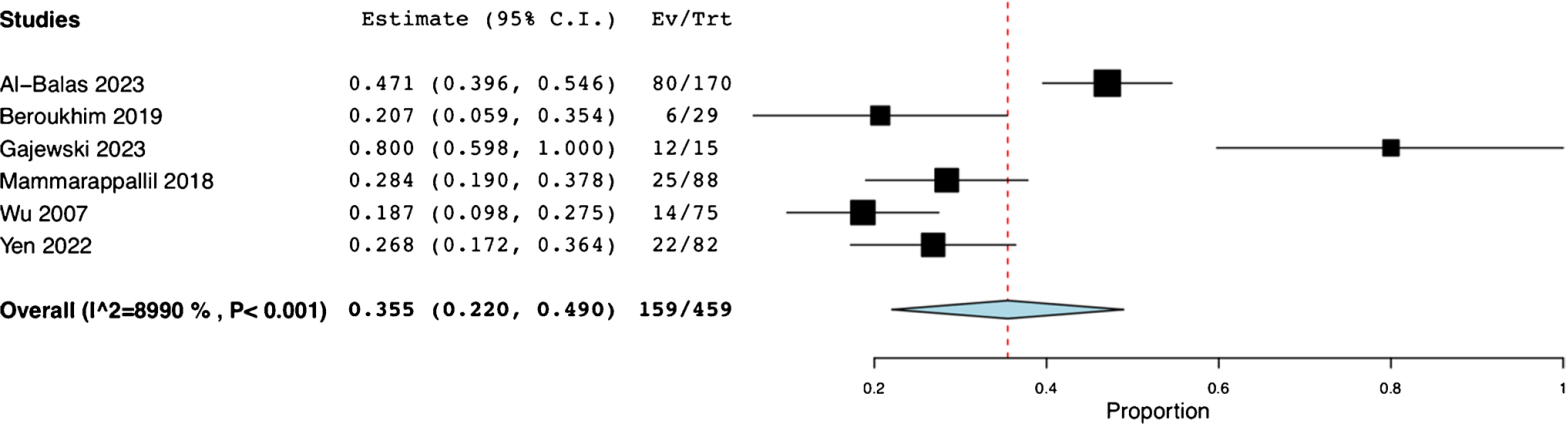
Pooled Rate of Positive Culture Yield – Overlying Skin Disease Avoidance Not Reported

**Fig. 4:**
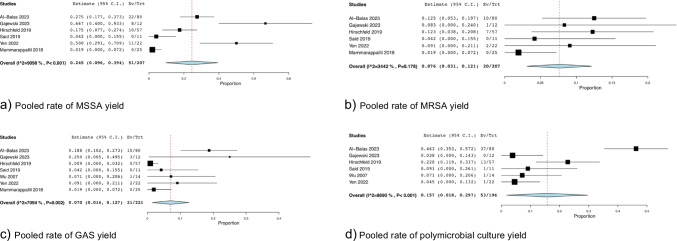
Causative Agent Yield (**a**) Pooled rate of MSSA yield (**b**) Pooled rate of MRSA yield (**c**) Pooled rate of GAS yield (**d**) Pooled rate of polymicrobial culture yield

All eight studies reported on bone culture results. Out of the total 722 samples sent, 227 samples were positive and 495 were negative [13–20]. The positive bone cultures accounted for 31.9% (95% CI: 0.222, 0.416; *I*^*2*^ = 87.83%) (Fig. 2b) while negative cultures were obtained in 68.1% of cases (95% CI: 0.584, 0.778; *I*^*2*^ = 87.83%) (Fig. 2c). We ran another analysis for positive culture yield rate, this time excluding the studies that specifically avoided ulcers and/or overlying cellulitis [16, 17], with a positive culture yield now pooled at 35.5% (95% CI: 0.220, 0.490; *I*^*2*^ = 89.90%) (Fig. 3). Six studies reported on Methicillin-Sensitive Staphylococcus Aureus (MSSA) and Methicillin-Resistant Staphylococcus Aureus (MRSA) yield [13, 15–17, 19, 20] at 24.5% (95% CI: 0.096, 0.394; *I*^*2*^ = 90.98%) (Fig. 4a) (N = 51) of the samples yielding MSSA and 7.6% (95% CI: 0.031, 0.121; *I*^*2*^ = 34.42%) (Fig. 4b) (N = 20) yielding MRSA cultures. Seven studies reported Group A Streptococcus (GAS) yield at 7.0% (95% CI: 0.014, 0.127; *I*^*2*^ = 70.94%) (Fig. 4c) (N = 21) [13, 15–20]. Two studies [14, 18] did not specify the exact distribution of yielded organisms but reported that most samples yielded Staphylococcus Aureus. Furthermore, polymicrobial culture was seen in 15.7% of cases (95% CI: 0.018, 0.297; *I*^*2*^ = 88.90%) (Fig. 4d) (N = 53) [13, 15–19].

**Fig. 5:**
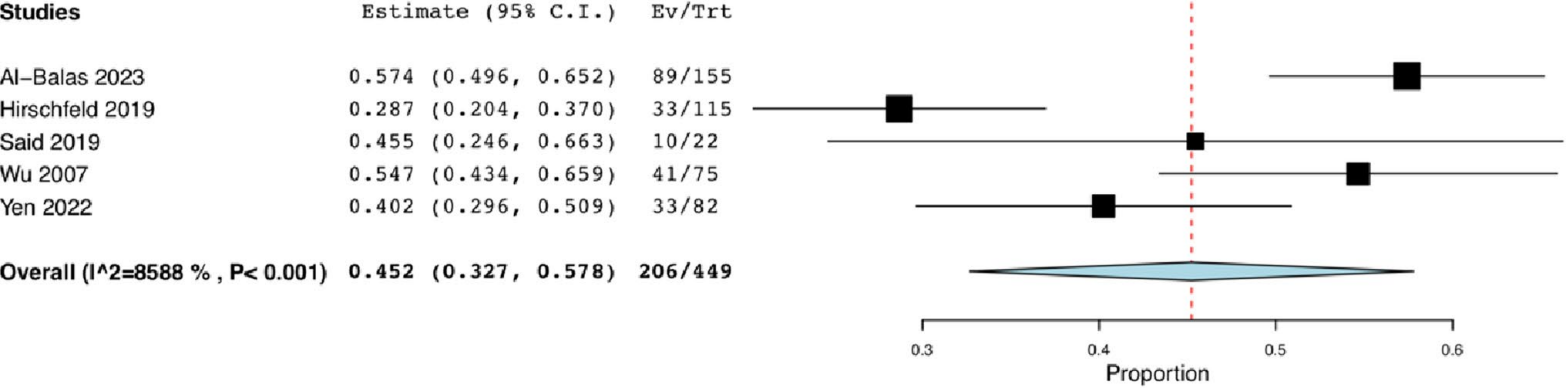
Pooled Rate of Positive Histological Sampling

Five studies reported on histopathological analysis [13, 16–19]. Of the 449 biopsies analyzed, positive samples for OM accounted for 45.2% of cases (95% CI: 0.327, 0.578; *I*^2^ = 85.88%) (Fig. 5) (N = 206). A total of 169 were negative for OM, and 74 were deemed inadequate [13, 16–19]. Furthermore, the presence of purulent fluid on aspiration was reported in two out of the eight studies in 13 out of 104 (12.5%) procedures [14, 18].

**Fig. 6:**
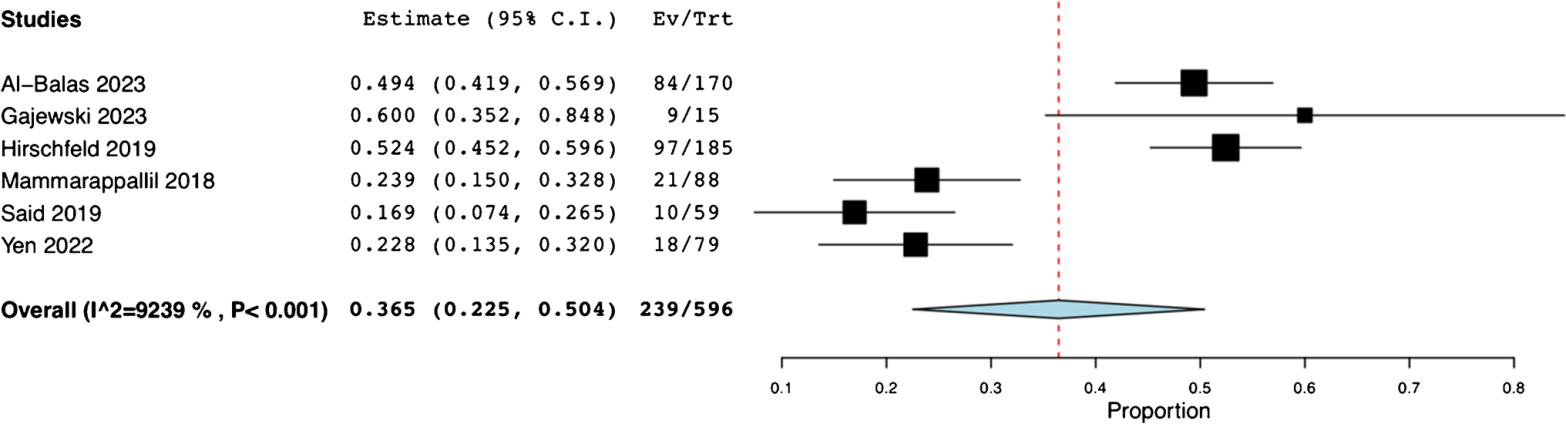
Pooled Rate of Post-Procedural Change in Management

Following the procedure, six studies reported a change in the management plan for 239 out of 596 patients. These represent 36.5% of total cases (95% CI: 0.225, 0.504; *I*^*2*^ = 92.39%) (Fig. 6) [13, 15–17, 19, 20]. Our definition for change in management was adapted from Hirschfeld et al.’s (2019) and includes any modification of antibiotics therapy including initiation, discontinuation, broadening or narrowing, as well as specific targeting of organisms identified on biopsy [16]. Four studies [13, 15, 17, 19] report 115 non-specific alterations to the management. Concerning the remaining 124 alterations, they were subclassified as follows: three studies [16, 17, 19] report the initiation of antibiotics in response to culture results in 30 cases (24.1%), while two studies [16, 20] report discontinuation of antibiotics in 10 cases (8.1%). Broadening of the antibiotic spectrum was reported in two studies [16, 20] for 18 patients (14.5%), while narrowing of the spectrum was performed in three studies [16, 19, 20] for 54 patients (43.5%). Targeting of a specific pathogen was undertaken in two studies [16, 17] for 12 cases (9.7%). No complications or mortality were reported from any of the included studies. The corresponding data is detailed in Table 4.

**Table 4:** Post-Procedural Outcomes

Study	Technical Success, n (%)	Inadequate Samples (n)	Aspiration of Purulent Fluid (n)	Samples Sent for Gram Staining (n)	Culture Results, Positive / Negative, n (%)	Organisms Yielded (n)	Samples Sent to Histology (n)	Histology Results, Positive / Negative, n (%)	Change in Management (n)
Al-Balas et al.	170 (100.0%)	0	NR	170	80 (47.1%) / 90 (52.9%)	GAS (15) MSSA (22) MRSA (10) Other* (33) Polymicrobial (37)	155	89 (57.4%) / 48 (30.9%) **	84
Beroukhim et al.	29 (100.0%)	0	3	29	6 (20.7%) / 23 (79.3%)	NR	NR	NR	NR
Gajewski et al.	15 (100.0%)	0	NR	15	12 (80.0%) / 3 (20.0%)	GAS (3) MSSA (8) MRSA (1)	NR	NR	9
Hirschfeld et al.	203 (100.0%)	0	NR	203	57 (28.1%) / 146 (71.9%)	MSSA (10) MRSA (7) Other* (27) Polymicrobial (13)	115	33 (28.7%) / 26 (22.6%) **	97
Mammarappallil et al.	NR	NR	NR	88	25 (28.4%) / 63 (71.6%)	NR	NR	NR	21
Said et al.	61 (98.4%)	1	NR	60	11 (18.3%) / 49 (81.7%)	Other* (11) Polymicrobial (1)	22	10 (45.5%) / 12 (54.5%)	10
Wu et al.	75 (100.0%)	0	10	75	14 (18.7%) / 61 (81.3%)	GAS (1) Other* (6) Polymicrobial (1)	75	41 (54.7%) / 34 (45.3%)	NR
Yen et al.	82 (100.0%)	0	NR	82	22 (26.8%) / 60 (73.2%)	GAS (2) MSSA (11) MRSA (2) Other* (5) Polymicrobial (1)	82	33 (40.2%) / 49 (59.8%)	18

*GAS* Group A Streptococcus, *MSSA* Methicillin susceptible Staphylococcus Aureus, *MRSA* Methicillin resistant Staphylococcus Aureus, *NR* Not reported, *n* Number

*Organisms that are not *GAS*, *MSSA* or *MRSA*, **The remaining samples were deemed inadequate or inconclusive

## Discussion

It is important to diagnose or refute suspected osteomyelitis. Obtaining accurate tissue samples for precise diagnosis may therefore significantly influence subsequent management decisions and treatment options for certain patients. Image-guided bone biopsies (IGBB) could play a pivotal role in this context, with this meta-analysis being the first to investigate its effectiveness in procuring tissue for microbiological and histological analyses from the appendicular skeleton. Here it is demonstrated that IGBB has a high technical success rate, supporting its capacity to procure accurate tissue samples for subsequent analysis. However, comparatively lower rates of positive bacterial cultures and histological findings were seen. Despite this, over a third of management plans were changed with the assistance of IGBB. As such, these findings carry implications for clinical practice, as they provide insight into the utility of IGBB in the investigation of osteomyelitis. In exploring and discussing these factors, this meta-analysis highlights the imperative for further exploration and refinement of diagnostic strategies tailored to the unique challenges posed by suspected osteomyelitis affecting the appendicular skeleton.

Our study demonstrated high rates of technical success. Strikingly, most of the studies included in our analysis reported a technical success rate of 100% [13–16, 18, 19]. These findings demonstrate the strong reliability of IGBs in the diagnostic setting of OM. Several factors contribute to the consistently high rates observed across studies. First, most of the included studies report that procedures were performed by specialist radiologists ensuring a high level of procedural efficiency [15, 17–20]. As such, the literature reflects these findings by consistently portraying percutaneous bone biopsies coupled with image guidance as straightforward procedures characterized by high rates of efficacy, further supporting the robustness of this diagnostic approach compared to other methods, such as blind bone biopsy [21]. Another point to consider is the role of pre-procedural imaging. All studies had undergone imaging prior to biopsy for localization of suspicious areas and subsequent targeting, with the most common modality being MRI (70%). MRI is known for its high sensitivity and specificity of OM detection [3, 22] and plays a significant role in detecting intraosseous abscesses and delineating extraosseous disease [23]. Adequate pre-procedural planning and subsequent targeting of suspicious zones are likely to increase the likelihood of obtaining adequate samples. Additional factors such as standardized guidelines and protocols attached to training, technique and equipment may have also contributed to the high technical rate across included studies.

Obtaining an adequate culture yield is important in establishing the optimal choice of antibiotic therapy [24]. When examining culture yield in our meta-analysis, we observed that rates of positive cultures reached 32%. There could have been multiple factors for this relatively low culture yield that warrants consideration. Firstly, the prevailing clinical recommendation is to typically discontinue antibiotics therapy 72 h before biopsy [25]. However, our analysis revealed that all eight studies included reported instances where patients were on antibiotics therapy before undergoing the IGBB procedure. Among these, two studies [13, 17] report discontinuation of antibiotics 24 h before the procedure, with their rate of positive cultures at 47% and 18%, respectively. The relationship between pre-procedural antibiotics and negative culture yield is well discussed in the literature, with the proposed hypothesis that empirical antibiotics therapy results in lower culture positivity [17]. Six of our included studies [13–17, 19] found no substantial correlation between pre-procedural antibiotics and culture yield. A previous meta-analysis by Chang et al. (2023) found no significant difference in microbiology sensitivity between patients who received antibiotics before biopsy and those who did not. It should be noted that this study was conducted on patients with vertebral osteomyelitis, and therefore comparisons with our non-vertebral osteomyelitis results should be made cautiously [26]. The results reported in a different meta-analysis by Crisologo et al. (2019) indicate that the impact of prebiopsy antibiotics on microbial culture yield varies significantly based on the type of osteomyelitis, biopsy methods, and study design, with no clear consensus in the literature. In patients with non-vertebral osteomyelitis, the results were similar to ours, with no significant difference found in culture yield between patients who were given antibiotics prior to procedure, and those who were not [27]. In contrast, Wu et al. (2007) reportedly observed a positive culture rate of 24% in patients where antibiotics were not discontinued 24 h before procedure, compared to 42% in patients where discontinuation was adhered to [18]. Beroukhim et al. (2019) proposed that cultures may be positive regardless of previous antibiotics therapy because of simultaneous ischemia due to the infection, or because of a vascular abnormality at the site of infection [14]. In considering all these factors, it should also be noted that Wu et al. was published in 2007, and that newer studies [13–17, 19, 26, 27] tend to observe no correlation between pre-procedural antibiotics and culture positivity, as previously mentioned. One potential way to combat this dilemma of pre-procedural antibiotics impacting the yield is the use of polymerase chain reaction (PCR), which is the last resort when other methods of pathogen identification has failed [28, 29]. Given the scarcity of studies that use PCR in this context, we encourage future studies to implement PCR to identify pathogens in those who have already been treated with antibiotics at the time of biopsy.

Another important consideration involves the presence of purulent fluid during aspiration. In the study by Wu et al. (2007), an observation was made that aspirations exceeding 2 mL of purulent fluid correlated with higher rates of positive cultures [18]. It is essential to note that two of our included studies did not observe this phenomenon [14, 19]. These studies also did not report the volume of aspirated fluid, and so it cannot be assumed that the amount was significant enough to observe a difference. These results suggest potential indication for IGBB in the case of osteomyelitis where intraosseous abscesses are identified on pre-operative imaging.

In relation to negative cultures, it has been suggested that the percutaneous bone technique increases the risk of false negatives due to several contributing factors. Firstly, the inherent complexity of reaching the target affected bone is amplified when overlying cellulitis is present. This complicates the biopsy procedure by either diverting needle towards the unaffected part of the bone, or by contaminating the deeper bone structures with microorganisms from the infected skin [25, 30, 31]. Furthermore, the complications posed by the overlying cellulitis is demonstrated by Hirschfeld et al. (2019), who observed the coexistence of overlying ulcers and cellulitis, in a substantial percentage of their patient cohort (42% and 81%, respectively) [16]. Despite efforts to exclude the ulcerated regions whenever feasible, as specified by Hirschfeld et al. (2019), the potential influence of the punctured skin's quality on the diagnostic yield in this population cannot be entirely discounted [16]. Our pooled analysis of positive culture yield for studies that did not specify avoiding passage through overlying skin disease was only slightly higher than the overall rate for positive culture yield. These results suggest however that attempting avoidance of ulcers and cellulitis is sensible, as this slight increase may reflect contamination of specimens.

Finally, our results revealed changes in treatment plan in 36.5% of cases. Notably, studies with lower rates of management changes coincided with those where rates of positive culture were the lowest [17, 19]. A previous study by Hoang et al. (2019) recorded post-procedural outcomes for 115 patients undergoing CT-guided biopsies for suspected OM and attributed no change in management to the results of the bone biopsies [22]. Although our results are not as extreme as Hoang et al. (2019), we believe that this correlation prompts critical reevaluation of the necessity and utility of IGBB and appropriate patient selection in suspected osteomyelitis of the appendicular skeleton. Additionally, as pointed out by Said et al. (2019), this benefit should be balanced out by the potential opportunity for complications that may arise, such as creating new wound tracks connecting overlying skin infection and bone tissue [17]. It should be noted that in two of the studies [13, 16], the number of patients for which a change in management was indicated exceeded the number of positive cultures. This point highlights the potential role of negative culture results in directing patient management. In fact, our results show that negative culture results played a more significant role in initiation (20 cases) [16, 17] rather than discontinuation (10 cases) [16, 20] of antibiotics regimen. And counterintuitively perhaps, 118 cases were reported where the same antibiotics regimen was maintained despite a negative culture yield [16, 17]. Further exploration into the intricate relationship between cultural yield and subsequent management strategies will enhance the precision and relevance of diagnostic algorithms in suspected OM.

This study had several limitations which warrant consideration. Firstly, the retrospective nature of the included studies introduces inherent challenges in preventing patient-selection bias. Additionally, the predominantly single-center focus of the studies introduces potential limitations in generalizing the results to a broader patient population. Concentration of studies within specific centers may introduce biases associated with regional variations in patient demographics, healthcare practices and diagnostic approaches. Moreover, all the included studies took place in the United States, which mitigates the reproducibility of the results to a different population. Although protocols are standardized across institutions, variabilities in technique and sampling equipment at the level of the operator may introduce variability in diagnostic accuracy. Furthermore, since some of our included studies are over 15 years old, biopsy devices and technique in the field of radiology have since evolved, which may have affected our outcomes. One study reported exclusively on the outcomes pertaining to their population with positive histological analysis (N = 41) which may limit reproducibility of the results [18]. Despite it being widely recognized as the gold standard for investigation of suspected OM [3, 22], MRI was used for pre-biopsy investigation in less than 80% of patients. Lastly, procedures such as IGBB, which require significant resources and specialized manpower, limit generalizability to areas where resources are scarcer. All the above limitations might have contributed to the high degree of heterogeneity seen in most of our findings which could have impacted the validity of the results. Future investigations, ideally through well-designed multicenter prospective studies with large sample sizes, as well as subgroup analyses focusing on more strictly defined anatomical regions (e.g., bones of the foot, pelvis etc.) and specific patient subsets (e.g., diabetic patients) should address these limitations to further refine the understanding of IGBB in non-vertebral OM.

For osteomyelitis (OM) of the appendicular skeleton, image-guided bone biopsy (IGBB) demonstrates promising rates of technical success for selected patients. The resulting culture yields lead to adjustments in almost a third of management plans with the implementation of IGBB. Overall, the integration of IGBB in the investigation and diagnostic pathway of OM needs to be carefully evaluated based on pre-procedural imaging. Additional studies comparing the diagnostic yield of IGBB under different imaging modalities, as well as in OM of the appendicular and axial skeletons may provide further guidance on its efficacy and utility.

## Supplementary Information

Below is the link to the electronic supplementary material.

Supplementary file1 (DOCX 18 KB)

Supplementary file2 (DOCX 78 KB)

Supplementary file3 (DOCX 733 KB)

Supplementary file4 (DOCX 407 KB)

## Data Availability

The data set used for this meta-analysis will be shared upon request from the study authors.

## Abbreviations

CT: Computed tomography
GAS: Group A streptococcus
IGBB: Image-guided bone biopsy
MRI: Magnetic resonance imaging
MRSA: Methicillin-resistant staphylococcus aureus
MSSA: Methicillin-susceptible staphylococcus aureus
OM: Osteomyelitis
PCR: Polymerase Chain Reaction
