# Supplementary material for: Yield and clinical impact of image-guided bone biopsy in osteomyelitis of the appendicular skeleton: a systematic review and meta-analysis
Source: Skeletal Radiol. 2024 Jul 30;54(3):481–92. doi: 10.1007/s00256-024-04764-7 (PMC11769862; doi:10.1007/s00256-024-04764-7)
Supplement: Supplementary file 1 — Supplementary file1 (DOCX 18 KB) [file 256_2024_4764_MOESM1_ESM.docx]

**Supplemental Figure A. Actual Search Strategy**

PubMed 181

**(Osteomyelitis)**

**AND**

**(Bone biopsy OR Osseous biopsy OR "biopsy, bone" OR "biopsy, vertebral" OR "bone puncture" OR "long bone biopsy" OR "pelvis biopsy" OR "skeletal biopsy" OR "spinal biopsy" OR "spine biopsy" OR "vertebral biopsy" OR "vertebral puncture" OR "bone biopsy" AND (Image-Guided) OR (image guided)) OR ((image guided biopsy) AND (bone)))**

("osteomyelities"[All Fields] OR "osteomyelitis"[MeSH Terms] OR "osteomyelitis"[All Fields] OR "osteomyelitides"[All Fields]) AND ((("Image-Guided"[All Fields] OR (("image"[All Fields] OR "image s"[All Fields] OR "imaged"[All Fields] OR "imager"[All Fields] OR "imager s"[All Fields] OR "imagers"[All Fields] OR "images"[All Fields] OR "imaging"[All Fields] OR "imaging s"[All Fields] OR "imagings"[All Fields]) AND ("guide"[All Fields] OR "guided"[All Fields] OR "guides"[All Fields] OR "guiding"[All Fields]))) AND ((("bone and bones"[MeSH Terms] OR ("bone"[All Fields] AND "bones"[All Fields]) OR "bone and bones"[All Fields] OR "bone"[All Fields]) AND ("biopsie"[All Fields] OR "biopsy"[MeSH Terms] OR "biopsy"[All Fields] OR "biopsied"[All Fields] OR "biopsies"[All Fields] OR "biopsy s"[All Fields] OR "biopsying"[All Fields] OR "biopsys"[All Fields] OR "pathology"[MeSH Subheading] OR "pathology"[All Fields])) OR (("bone and bones"[MeSH Terms] OR ("bone"[All Fields] AND "bones"[All Fields]) OR "bone and bones"[All Fields] OR "osseous"[All Fields]) AND ("biopsie"[All Fields] OR "biopsy"[MeSH Terms] OR "biopsy"[All Fields] OR "biopsied"[All Fields] OR "biopsies"[All Fields] OR "biopsy s"[All Fields] OR "biopsying"[All Fields] OR "biopsys"[All Fields] OR "pathology"[MeSH Subheading] OR "pathology"[All Fields])) OR "biopsy bone"[All Fields] OR "biopsy vertebral"[All Fields] OR "bone puncture"[All Fields] OR "long bone biopsy"[All Fields] OR "pelvis biopsy"[All Fields] OR "skeletal biopsy"[All Fields] OR "spinal biopsy"[All Fields] OR "spine biopsy"[All Fields] OR "vertebral biopsy"[All Fields] OR "vertebral puncture"[All Fields])) OR (("image guided biopsy"[MeSH Terms] OR ("Image-Guided"[All Fields] AND "biopsy"[All Fields]) OR "image guided biopsy"[All Fields] OR ("image"[All Fields] AND "guided"[All Fields] AND "biopsy"[All Fields]) OR "image guided biopsy"[All Fields]) AND ("bone and bones"[MeSH Terms] OR ("bone"[All Fields] AND "bones"[All Fields]) OR "bone and bones"[All Fields] OR "bone"[All Fields])))

EMBASE 216

Embase

Session Results

.......................................................

No. Query Results Results Date

#11. #1 AND #10 216 5 Oct 2023

#10. #4 OR #9 2,385 5 Oct 2023

#9. #7 AND #8 318 5 Oct 2023

#8. 'image guided' 42,697 5 Oct 2023

#7. #5 OR #6 15,292 5 Oct 2023

#6. 'biopsy, bone' OR 'biopsy, vertebral' OR 'bone 15,292 5 Oct 2023

puncture' OR 'long bone biopsy' OR 'long bones

biopsy' OR 'pelvis biopsy' OR 'skeletal biopsy'

OR 'spinal biopsy' OR 'spine biopsy' OR 'vertebra

biopsy' OR 'vertebra puncture' OR 'vertebral

biopsy' OR 'vertebral puncture' OR 'bone biopsy' OR “Osseous biopsy”

#5. 'bone biopsy'/exp 13,392 5 Oct 2023

#4. #2 AND #3 2,283 5 Oct 2023

#3. 'bone'/exp OR bone 2,194,865 5 Oct 2023

#2. 'image guided biopsy'/exp OR 'image guided 21,384 5 Oct 2023

biopsy'

#1. 'osteomyelitis'/exp OR osteomyelitis 59,164 5 Oct 2023

Search Name:

Date Run: 05/10/2023 11:43:17

Comment:

Cochrane 0

ID Search Hits

#1 MeSH descriptor: [Osteomyelitis] explode all trees 207

#2 (Osteomyelitis):ti,ab,kw (Word variations have been searched) 684

#3 MeSH descriptor: [Image-Guided Biopsy] explode all trees 357

#4 ("image guided biopsy"):ti,ab,kw (Word variations have been searched) 262

#5 #3 OR #4 443

#6 MeSH descriptor: [Bone and Bones] explode all trees 17802

#7 (bone):ti,ab,kw (Word variations have been searched) 66226

#8 #6 OR #7 75588

#9 #5 AND #8 4

#10 ('biopsy, bone' OR 'biopsy, vertebral' OR 'bone puncture' OR 'long bone biopsy' OR 'long bones biopsy' OR 'pelvis biopsy' OR 'skeletal biopsy' OR 'spinal biopsy' OR 'spine biopsy' OR 'vertebra biopsy' OR 'vertebra puncture' OR 'vertebral biopsy' OR 'vertebral puncture' OR 'bone biopsy' OR 'Osseous biopsy'):ti,ab,kw (Word variations have been searched) 5406

#11 ('image guided'):ti,ab,kw (Word variations have been searched) 8188

#12 #10 AND #11 124

#13 #9 OR #12 124

#14 #1 OR #2 693

#15 #13 AND #14 0

Scopus 88

( TITLE-ABS-KEY ( osteomyelitis ) ) AND ( TITLE-ABS-KEY ( "image guided" ) ) AND ( TITLE-ABS-KEY ( "biopsy, bone" OR "biopsy, vertebral" OR "bone puncture" OR "long bone biopsy" OR "pelvis biopsy" OR "skeletal biopsy" OR "spinal biopsy" OR "spine biopsy" OR "vertebral biopsy" OR "vertebral puncture" OR "bone biopsy" OR “Osseous biopsy” ) )

Web of Science

# Web of Science Search Strategy (v0.1)

# Database: Web of Science Core Collection

# Entitlements:

- WOS.IC: 1993 to 2023

- WOS.CCR: 1985 to 2023

- WOS.SCI: 1900 to 2023

- WOS.AHCI: 1975 to 2023

- WOS.BHCI: 2005 to 2023

- WOS.BSCI: 2005 to 2023

- WOS.ESCI: 2005 to 2023

- WOS.ISTP: 1990 to 2023

- WOS.SSCI: 1900 to 2023

- WOS.ISSHP: 1990 to 2023

# Searches:

1: TS=(osteomyelitis) Date Run: Thu Oct 05 2023 19:31:53 GMT+1000 (Australian Eastern Standard Time) Results: 27803

2: TS=("image guided") Date Run: Thu Oct 05 2023 19:32:17 GMT+1000 (Australian Eastern Standard Time) Results: 25124

3: TS=(“biopsy, bone” OR “biopsy, vertebral” OR “bone puncture” OR “long bone biopsy” OR “pelvis biopsy” OR “skeletal biopsy” OR “spinal biopsy” OR “spine biopsy” OR “vertebral biopsy” OR “vertebral puncture” OR “bone biopsy” OR “Osseous biopsy”) Date Run: Thu Oct 05 2023 19:32:50 GMT+1000 (Australian Eastern Standard Time) Results: 2844

4: #1 AND #2 AND #3 Date Run: Thu Oct 05 2023 19:33:31 GMT+1000 (Australian Eastern Standard Time) Results: 21

Cinahl 6

| **#** | **Query** | **Limiters/Expanders** | **Last Run Via** | **Results** |
| --- | --- | --- | --- | --- |
| S6 | S3 AND S4 AND S5 | Expanders - Apply equivalent subjects Search modes - Boolean/Phrase | Interface - EBSCOhost Research Databases Search Screen - Advanced Search Database - CINAHL Complete | 6 |
| S5 | "image guided" | Expanders - Apply equivalent subjects Search modes - Boolean/Phrase | Interface - EBSCOhost Research Databases Search Screen - Advanced Search Database - CINAHL Complete | 4,052 |
| S4 | “biopsy, bone” OR “biopsy, vertebral” OR “bone puncture” OR “long bone biopsy” OR “pelvis biopsy” OR “skeletal biopsy” OR “spinal biopsy” OR “spine biopsy” OR “vertebral biopsy” OR “vertebral puncture” OR “bone biopsy” OR “Osseous biopsy” | Expanders - Apply equivalent subjects Search modes - Boolean/Phrase | Interface - EBSCOhost Research Databases Search Screen - Advanced Search Database - CINAHL Complete | 565 |
| S3 | S1 OR S2 | Expanders - Apply equivalent subjects Search modes - Boolean/Phrase | Interface - EBSCOhost Research Databases Search Screen - Advanced Search Database - CINAHL Complete | 6,397 |
| S2 | (MM "Osteomyelitis") | Expanders - Apply equivalent subjects Search modes - Boolean/Phrase | Interface - EBSCOhost Research Databases Search Screen - Advanced Search Database - CINAHL Complete | 3,236 |
| S1 | Osteomyelitis | Expanders - Apply equivalent subjects Search modes - Boolean/Phrase | Interface - EBSCOhost Research Databases Search Screen - Advanced Search Database - CINAHL Complete | 6,397 |
