# Supplementary material for: Yield and clinical impact of image-guided bone biopsy in osteomyelitis of the appendicular skeleton: a systematic review and meta-analysis
Source: Skeletal Radiol. 2024 Jul 30;54(3):481–92. doi: 10.1007/s00256-024-04764-7 (PMC11769862; doi:10.1007/s00256-024-04764-7)
Supplement: Supplementary file 2 — Supplementary file2 (DOCX 78 KB) [file 256_2024_4764_MOESM2_ESM.docx]

Studies from databases/registers **(n = 370)**

References from other sources **(n = 0)**

Citation searching (n = 0)

Grey literature (n = 0)

**Identification**

Included studies ongoing **(n = 0)**

Studies awaiting classification **(n = 0)**

Studies included in review **(n = 8)**

Studies excluded **(n = 344)**

Studies not retrieved **(n = 0)**

Studies assessed for eligibility **(n = 16)**

Studies sought for retrieval **(n = 16)**

Studies screened **(n = 360)**

Studies excluded **(n = 8)**

Wrong intervention (n = 2)

Wrong study design (n = 1)

Conference abstract (n = 4)

Wrong patient population (n = 1)

References removed **(n = 10)**

Duplicates identified manually (n = 2)

Duplicates identified by Covidence (n = 8)

Marked as ineligible by automation tools (n = 0)

Other reasons (n = 0)

**Screening**

**Included**
