# Supplementary figures and images for: Yield and clinical impact of image-guided bone biopsy in osteomyelitis of the appendicular skeleton: a systematic review and meta-analysis
Source: Skeletal Radiol. 2024 Jul 30;54(3):481–92. doi: 10.1007/s00256-024-04764-7 (PMC11769862; doi:10.1007/s00256-024-04764-7)

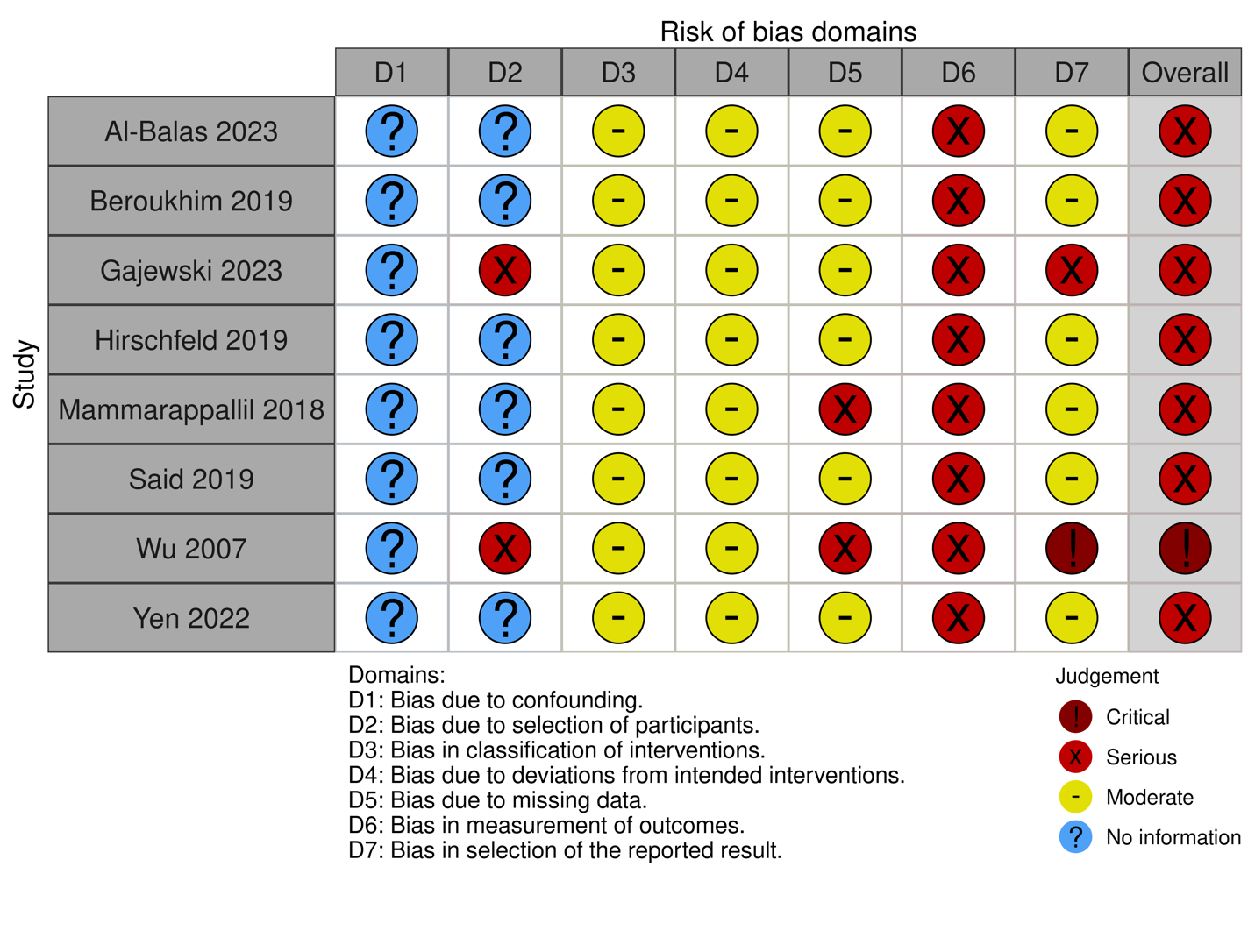


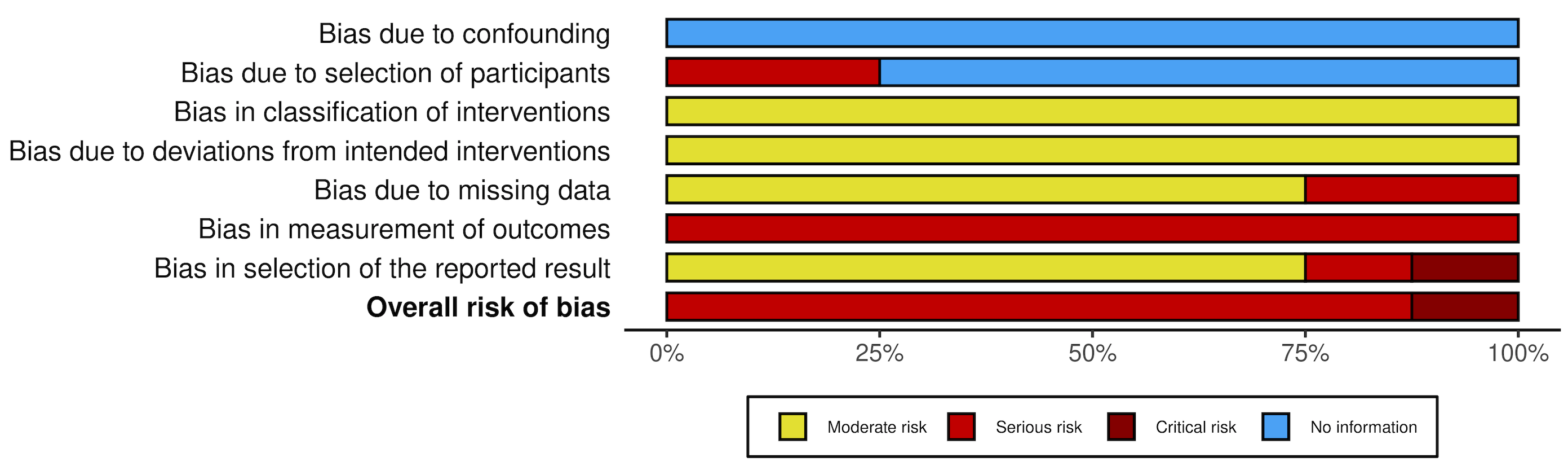


**Supplementary Figure C. ROBINS-I Tool**

Supplement: Supplementary file 3 — Supplementary file3 (DOCX 733 KB) [file 256_2024_4764_MOESM3_ESM.docx]
