# Supplementary material for: Yield and clinical impact of image-guided bone biopsy in osteomyelitis of the appendicular skeleton: a systematic review and meta-analysis
Source: Skeletal Radiol. 2024 Jul 30;54(3):481–92. doi: 10.1007/s00256-024-04764-7 (PMC11769862; doi:10.1007/s00256-024-04764-7)
Supplement: Supplementary file 4 — Supplementary file4 (DOCX 407 KB) [file 256_2024_4764_MOESM4_ESM.docx]

**Supplementary Fig. D. Funnel Plots of Included Studies**

1. **** Pooled Rate of Pre-Procedural Antibiotics - Funnel Plot of Included Studies
2. Technical and Culture Yield Outcomes - Funnel Plot of Included Studies
3. Pooled Rate of Technical Success - Funnel Plot of Included Studies
4. Pooled Rate of Positive Culture Yield - Funnel Plot of Included Studies

1. Pooled Rate of Negative Culture Yield - Funnel Plot of Included Studies

1. Pooled Rate of Positive Culture Yield – Overlying Skin Disease Avoidance Not Reported - Funnel Plot of Included Studies

1. Causative Agent Yield - Funnel Plot of Included Studies
2. Pooled Rate of MSSA Yield b) Pooled Rate of MRSA Yield - Funnel Plot of Included Studies


 c) Pooled Rate of GAS Yield d) Pooled Rate of Polymicrobial Cultures - Funnel Plot of Included Studies

1. Pooled Rate of Positive Histological Sampling - Funnel Plot of Included Studies

1. Pooled Rate Post-Procedural Change in Management - Funnel Plot of Included Studies
